# Microstructural changes in compressed nerve roots treated by percutaneous transforaminal endoscopic discectomy in patients with lumbar disc herniation

**DOI:** 10.1097/MD.0000000000005106

**Published:** 2016-10-07

**Authors:** Weifei Wu, Jie Liang, Ying Chen, Aihua Chen, Bin Wu, Zong Yang

**Affiliations:** a Department of Orthopedics; b Department of Nephrology; c Department of Radiology, the People's Hospital of Three Gorges University· the First People's Hospital of Yichang, Hubei, China.

**Keywords:** diffusion tensor imaging, herniated disc, microstructural properties of nerve roots, percutaneous transforaminal endoscopic discectomy

## Abstract

To investigate the microstructural changes in compressed nerves using diffusion tensor imaging (DTI) of herniated disc treated with percutaneous transforaminal endoscopic discectomy.

Diffusion tensor imaging has been widely used to visualize peripheral nerves, and the microstructure of compressed nerve roots can be assessed using DTI. However, the microstructural changes after surgery are not well-understood in patients with lumbar disc herniation.

Thirty-four consecutive patients with foraminal disc herniation affecting unilateral sacral 1 (S1) nerve roots were enrolled in this study. DTI with tractography was performed on S1 nerve roots before and after surgery. The mean fractional anisotropy (FA) and apparent diffusion coefficient values were calculated from tractography images.

In compressed nerve roots, the FA value before surgery was significantly lower than that after surgery (*P* = 0.000). A significant difference in FA values was found between the compressed and normal sides before surgery (*P* = 0.000). However, no significant difference was found between the compressed and normal sides after surgery (*P* = 0.057). A significant difference in apparent diffusion coefficient values was found before and after surgery at the compressed side (*P* = 0.023). However, no significant difference was found between the compressed and normal sides after surgery (*P* = 0.203).

We show that the diffusion parameters of compressed nerve roots were not significantly different before and after percutaneous transforaminal endoscopic discectomy, indicating that the microstructure of the nerve root recovered after surgery.

## 1 Introduction

Lumbar disc herniation (LDH) resulting in the compression of nerve roots is not uncommon. Surgical treatment is necessary in patients with leg and hip pain, and numbness. With the development of surgical techniques, most patients with LDH can be treated with minimally invasive surgery, especially percutaneous transforaminal endoscopic discectomy.^[1,2]^

Magnetic resonance imaging (MRI) provides valuable information regarding the location and size of disc herniation, and its mass impact on proximal nerves, and hence, is often used to diagnose disc herniation.^[3]^ However, inconsistencies between the symptoms and the degree of nerve root compression seen on conventional MRI are repeatedly found. Some studies have found that symptom-free people can demonstrate nerve root compression.^[2,3]^ Therefore, it is difficult to understand the cause of the pain and numbness, either before or after surgery, with conventional MRI alone. Moreover, MRI cannot provide a quantitative assessment of preoperative nerve root damage or postoperative nerve root recovery. Diffusion tensor imaging (DTI), which is a qualitative tool used to identify the path of nerves that is not readily observed by conventional MRI methods, has been extensively used in the central nervous system.^[4,5]^ DTI of molecular water movement has been proven to be a valuable method for evaluating microstructural changes in nerves.^[6]^ The diffusion data can be used to determine quantitative diffusion values, such as apparent diffusion coefficient (ADC) and fractional anisotropy (FA), which reflect the directionality of molecular diffusion; high FA values indicate anisotropic diffusion and low FA values indicate more isotropic diffusion.^[7]^ Our previous study found that DTI, performed with a 1.5-T MRI scanner, can evaluate the physiopathology of compressed nerve roots in patients with LDH.^[8]^ Although a close relationship between clinical symptoms and DTI parameters in patients with LDH has been strongly suggested, the microstructural functional recovery of compressed nerves after treating the herniated disc remains unknown. The aim of our study was to investigate the microstructural changes as evaluated by DTI in compressed nerves after treating the herniated disc.

## 2 Methods

### 2.1 Subjects

The approval of the Three Gorges University review boards was obtained before conducting the present study. All subjects provided written informed consent, allowing their clinical data to be used for research. LDH patients presented to the orthopedic inpatient department between May 2015 and December 2015 were included. Only patients aged 25 to 50 years were included in our prospective study, because, in clinical practice, patients with LDH over the age of 50 years are often treated with a pedicle screw, which has a serious influence on DTI scanning. The other inclusion criteria were unilateral sacral 1 (S1) LDH, radicular pain, and computed tomography or MRI evidence of disc herniation compressing the nerve root. Exclusion criteria were a previous history of trauma or surgery (not limited to the spine), osteoarthritis, hypertension, diabetes, or neurological disease, or a contraindication for MRI, such as pregnancy or metallic implants. All patients were treated with standard percutaneous transforaminal endoscopic discectomy, and all operations were performed by the same surgeon (JL).

### 2.2 MRI

**Figure 1:**
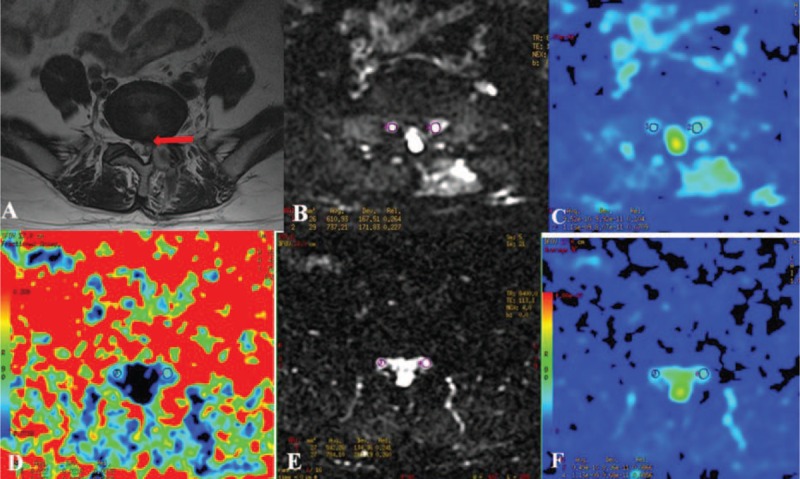
The FA and ADC measurements before and after surgery in patients with unilateral S1 disc herniation. The S1 nerve root was compressed on the left side (A). Bilateral nerve roots were assessed by DTI before (B) and after surgery (D). The ADC (C and F) and FA (E) values at the most compressed part are measured using the manual setting of the ROIs before and after surgery. B, FA before surgery (left side: 0.187, right side: 0.262); E, FA after surgery (left side: 0.242, right side: 0.259); C, ADC (10^−3^ mm^2^/s) before surgery (left side: 1.451, right side: 1.311); F, ADC (10^−3^ mm^2^/s) after surgery (left side: 1.286, right side: 1.372). ADC = apparent diffusion coefficient, DTI = diffusion tensor imaging, FA = fractional anisotropy, S1 = sacral 1.

Magnetic resonance imaging scans were performed using a single 1.5-T GE system (GE Healthcare, Chalfont St. Giles, UK) on the day of study enrollment and at least 1 month after surgery. We used a 6 AQ6-element phased array spine coil, and images were acquired with patients in the supine position. A standard MRI protocol was performed before and after surgery, which included both T1-weighted turbo spin echo (TSE) (repetition time [TR], 660 ms; echo time [TE], 9.5 ms; number of excitations [NEX], 1; field of view (FOV), 380 × 380 mm; matrix, 128 × 128; slice count, 12; slice thickness, 5 mm; slice gap, 0.4 mm; acquisition time, 2 minutes 53 seconds) and T2-weighted TSE (TR, 2960 ms; TE, 70 ms; NEX, 2; FOV, 380 × 380 mm; matrix, 512 × 512; slice count, 12; slice thickness, 4 mm; slice gap, 0.4 mm; acquisition time, 3 minutes 21 seconds) sequences, imaging the lumbar spine in the sagittal plane. A T2-weighted TSE (TR, 5680 ms; TE, 123 ms; FOV, 200 × 200 mm; matrix, 512 × 512; NEX, 2; slice count, 30; slice thickness, 3 mm; slice gap, 0; acquisition time, 3 minutes 40 seconds) sequence was performed in the axial plane, exploring the last 2 mobile levels of the lumbar spine. In addition to these sequences, a single-shot echo-planar spin-echo DTI sequence was performed in the axial plane of the L4-to-S1 interbody spaces with the following parameters: TR, 8400 ms; TE, 89.1 ms; FOV, 240 × 240 mm; matrix, 128 × 128; NEX, 4; slice count, 23; slice thickness, 3 mm; slice gap, 0; *b* value, 900 s/mm^2^; motion probing gradients applied in 15 directions; acquisition time, 9 minutes 01 second. The range of DTI scanning in this study was from the lower half of L4 to S1, which covered all of S1 nerve roots (Fig. 1).

### 2.3 Data analysis

**Figure 2:**
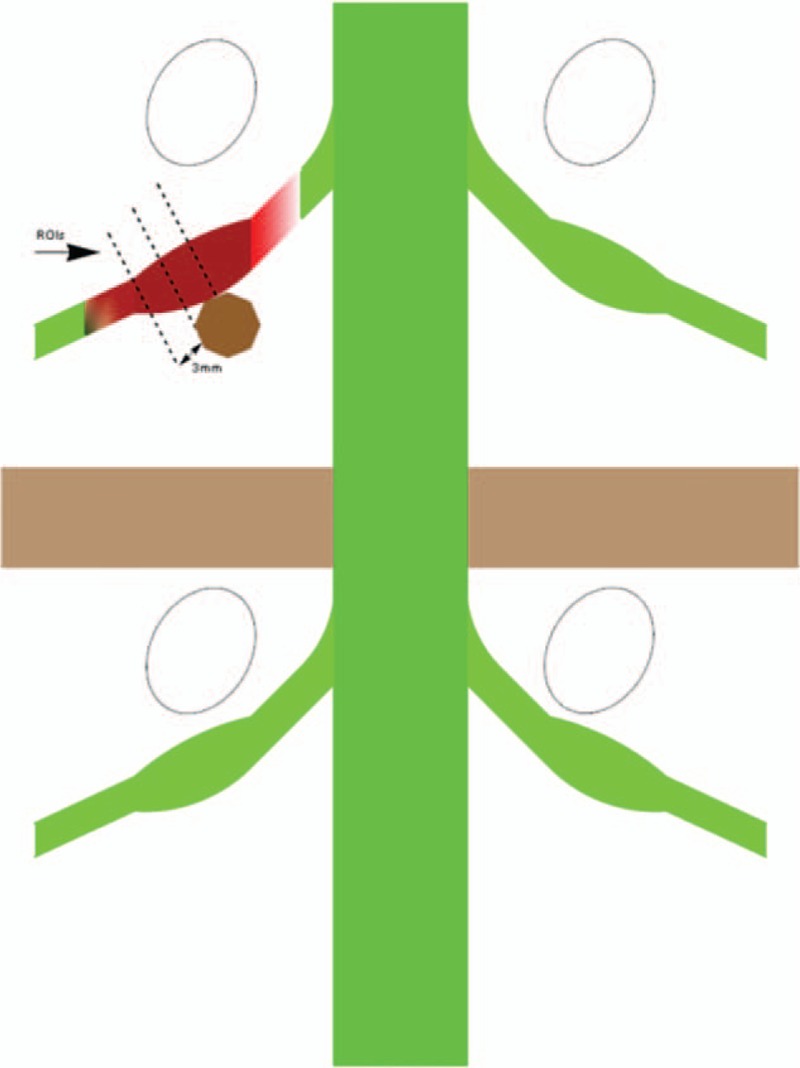
Diagram showing that the regions of interest (ROIs) were placed at 3 levels at 3-mm intervals proximal to the site of compression of the nerve by disc herniation on the axial image of the calculated.

All MRI scans were reviewed and agreed upon by 2 radiologists blinded to the clinical data; both had significant experience in interpreting imaging of the spine. Image analysis was independently performed for each participant immediately after image acquisition for qualitative assessment and then for data extraction. Neurography was obtained using the diffusion volume (*b* value, 900 s/mm^2^), which was visualized as a maximum intensity projection, to avoid images with obvious artifacts. Anatomical axial T2 and DTI images were merged to allow a better visualization of the different anatomic spaces. FA and ADC maps were generated using FiberViewer (http://www.ia.unc.edu/dev) software. Measurements were focused on the site of the compression, and in the same area on the contralateral root, before and after the surgery. The relative location of the region of interest (ROI) before the surgery was marked relative to the vertebral body and pedicle on both the compressed and contralateral sides in each patient. The postoperative ROI was identified relative to the preoperative marked sites (Fig. 1). The ROI was circular with a diameter of 3 voxels; the averaged volume of the ROI at compressed nerve roots was the same as that of the contralateral side. The ROIs were placed at 3 levels of the compressed nerve root as shown in Fig. 2. The size of ROIs, ranging from 20 to 45 mm^2^, was selected to accurately include the respective nerve roots while limiting the presence of other tissues to avoid partial volume effects when the mean FA and ADC were calculated. The diffusion tensor fields were diagonalized to obtain eigenvalues and eigenvectors for each voxel. The eigenvector associated with the largest eigenvalue was used to represent the main direction.

### 2.4 Statistical analysis

The statistical analysis was conducted using a standard SPSS 16.0 (SPSS Institute, Chicago, IL) software package. Data are expressed as the mean ± standard deviation. Since the data in our study were continuous and normally distributed, an independent-samples *t* test was performed to compare the compressed and normal sides before and after surgery at the same spinal segment. *P* < 0.05 was considered significant.

## 3 Results

**Figure 3:**
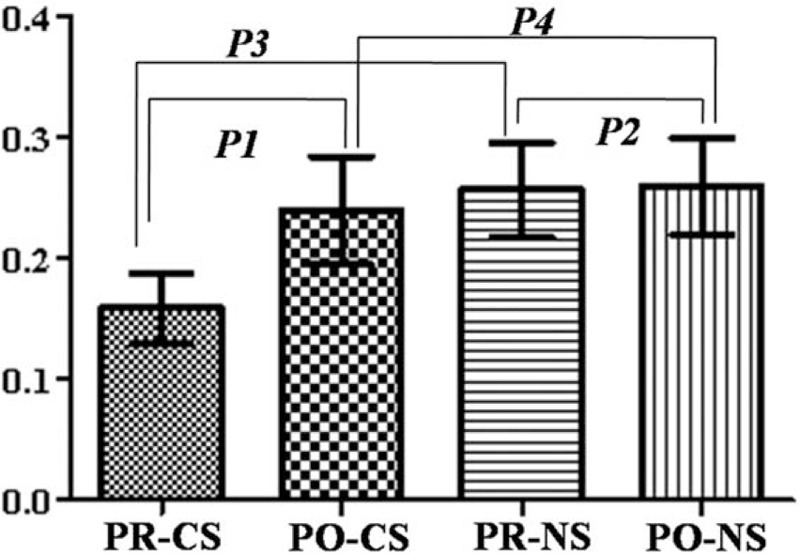
The FA value change in compressed nerve roots before and after surgery in patients with unilateral S1 disc herniation (*P1* = 0.000, *P2* = 0.744, *P3* = 0.000, *P4* = 0.057). CS = compressed side, NS = normal side, PO = postoperation, PR = preoperation, S1 = sacral 1.

**Figure 4:**
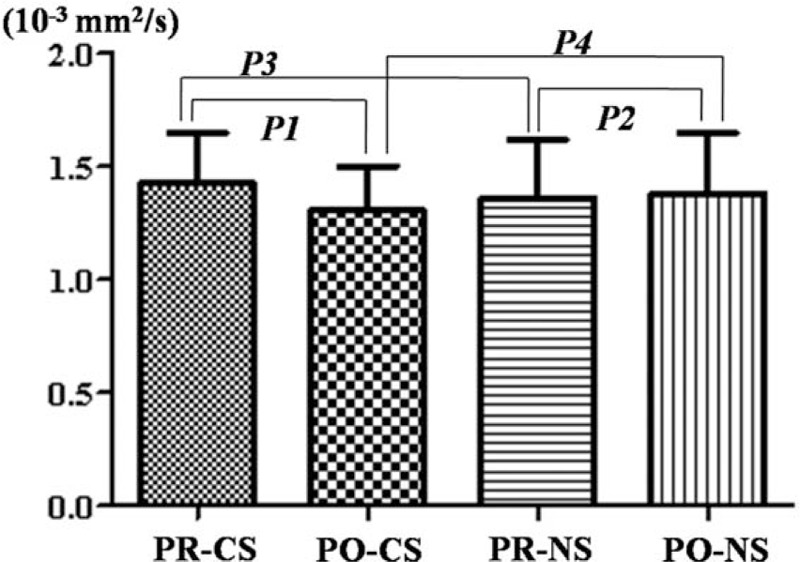
The ADC value change in compressed nerve roots before and after surgery in patients with unilateral S1 disc herniation (*P1* = 0.023, *P2* = 0.773, *P3* = 0.277, *P4* = 0.203). CS = compressed side, NS = normal side, PO = postoperation, PR = preoperation, S1 = sacral 1.

A total of 34 consecutive patients with LDH (mean age, 35.2 ± 7.4 years) were included in the present study. The sample comprised of 20 males and 14 females. The second DTI scan was performed at least 1 month after the surgery (mean duration: 2.5 months, range: 1.3–4.4 months). The FA value of compressed nerve roots was 0.160 (range: 0.111–0.250) and 0.240 (range: 0.187–0.341) before and after surgery, respectively (Fig. 3). A significant difference was found between these values before and after surgery on the compressed side (*P* = 0.000). The FA value of the normal nerve roots was 0.257 (range: 0.188–0.341) and 0.260 (range: 0.180–0.320) before and after surgery, respectively. No significant difference was found between these values before and after surgery on the normal side (*P* = 0.744). A significant difference in FA values was found between the compressed and normal sides before surgery (*P* = 0.000), but not after surgery (*P* = 0.057). The ADC value (×10^−3^ mm^2^/s) of the compressed nerve roots was 1.427 (range: 1.000–1.890) and 1.308 (range: 0.908–1.792) before and after surgery, respectively (Fig. 4). A significant difference was found between these values before and after surgery on the compressed side (*P* = 0.023). The ADC value of normal nerve roots was 1.363 (range: 0.789–1.799) and 1.381 (range: 0.752–1.772) before and after surgery, respectively. No significant difference was found between these values before and after surgery on the normal side (*P* = 0.773). No significant difference in ADC values was found between the compressed and normal sides both before and after surgery (*P* = 0.277 and 0.203, respectively).

## 4 Discussion

The resolution or improvement of symptoms such as pain and numbness is the most important concern for patients with LDH after surgery. However, the symptoms may persist for a short time after surgery in some patients. Microstructural improvement in the nerve roots may be seen before any change in symptoms. Therefore, assessment of microstructural changes in compressed nerve roots is important in clinical practice to predict potential improvement of clinical symptoms. DTI can provide an accurate assessment of the damage in compressed nerve roots.^[6,9–11]^ This knowledge allows a better understanding of how tissue damage causes clinical deficits and why changes in clinical symptoms occur later than microstructural changes after surgery. This information is not available with conventional MRI and other technologies.

The FA values reflect the size of the anisotropy of the analyzed structure, evaluated by taking advantage of the improved directional evaluation of water diffusivity in abnormal areas. Recent studies^[6,9,11,12]^ have reported that FA and ADC values might be a potential tool to evaluate the severity of nerve entrapment and diagnose the lumbar nerve entrapment. The mean FA values in entrapped nerve roots were significantly lower than those in intact nerve roots. In patients with sciatica, FA values were decreased in compressed nerve roots and they declined from the proximal-to-distal region along the compressed nerve tracts. The mean FA values were more sensitive and specific than MRI for differentiating compressed nerve roots.^[10,13]^ Our results showed a significant decrease in mean FA values compared with the contralateral nerve root, consistent with our previous study.^[8]^ These results indicate that in patients with clinical symptoms, diffusion in the tissue became more isotropic. Mechanical compression and chemical irritation in nerve roots can lead to the reduction of blood flow and ischemia. The balance of blood–nerve barrier will disorder, and then subperineurial or intraneural edema will arise. Assorted histological changes of nerve roots including the demyelination of nerve fibers, endoneurial fibrosis, and Wallerian degeneration can gradually occur.^[14,15]^ Due to the distance between axons and axon fascicles caused by microstructural changes increases, water diffusion along the nerve will be altered. Diffusion perpendicular to the largest eigenvalue will increase, which results in a decrease of FA values that associates with the axonal degeneration.^[16]^ Histological data from mouse models revealed that FA values positively are associated with the total number of regenerating axons.^[17]^

The ADC is a scalar value reflecting molecular diffusivity under motion restriction. There is some controversy regarding ADC in compressed nerve roots. Studies have found that ADC is significantly higher in compressed nerve roots than in contralateral uncompressed nerve roots.^[18–20]^ However, other studies have found that the ADC value of compressed nerve roots is similar to that of normal nerve roots in LDH patients.^[9,10]^ The present study found that in compressed nerve roots, the ADC value before surgery was significantly higher than that after surgery. However, there was no noticeable difference between the 2 sides both before and after surgery. The cause of the elevated ADC values is uncertain. Nerve root compression by disc herniation might lead to ischemia, edema, atrophy, anoxemia, and cellular membrane injury that could increase cellular membrane penetrability, potentially causing a disturbance of water molecular movement, and also decreased perfusion.

In patients with LDH who do not achieve a good recovery with conservative treatment, surgical interventions that include microendoscopic discectomy, discectomy and instrumentation with pedicle screws, and percutaneous transforaminal endoscopic discectomy should be considered.^[1,21]^ However, the microstructural improvement of compressed nerve roots is highly important to ensure surgical success. Although DTI is used widely for peripheral nerves, such as nerve roots in the lumbar region, those assessments have typically focused on the normal or preoperative population.^[9–13,20]^ Whether the microstructure of compressed nerve roots change after surgery in patients with LDH has not yet been examined. In clinical practice, after surgery, the quality of DTI in re-examination is often diminished by the metal instrument fixed on the vertebral pedicles and surrounding tissue edema. Therefore, in this study, the surgery was performed using a percutaneous transforaminal endoscopic approach and the second DTI scan was performed at least 1 month after surgery. We show that both FA and ADC values before surgery were significantly different from their corresponding values after surgery on the compressed side. However, after surgery, no noticeable difference was found between the compressed and normal sides in either FA or ADC values. The mechanism of DTI recovery might have several aspects. After surgery, the herniated disc was removed, which resulted in a gradual improvement in subperineurial and intraneural edema, blood flow, and ischemia of compressed nerve roots. Microstructural changes before surgery, such as demyelination of the nerve fibers, Wallerian degeneration, and endoneurial fibrosis, might partly or mostly recover. Therefore, water diffusion along the nerve returns to normal or near normal.

There are several limitations in the present study. First, the total number of patients in the study was small, and further prospective analyses with a larger number of patients are required to confirm its reproducibility. Second, placing the same ROIs before and after surgery might have an effect on the DTI values. Third, the clinical relationship between ADC and FA after surgery is still unclear. A comprehensive evaluation using DTI parameters would be more desirable.

## 5 Conclusions

This preliminary study demonstrated that DTI of the sacral nerves before and after surgery is possible. Significant changes in the diffusion parameters were found in compressed sacral nerves in patients with LDH and leg pain after surgery, indicating that the microstructure of the nerve root had recovered.

## Acknowledgment

The authors thank Dan Li very much for MRI technique support.
